# “It’s important to foster open discussion about the topic”: development, implementation, and evaluation of an ethics of abortion independent learning module for second year medical students

**DOI:** 10.1186/s12978-023-01686-w

**Published:** 2023-09-25

**Authors:** Catherine A. McCarty, Sarah L. Hutto, Aubie K. Shaw

**Affiliations:** 1 Department of Family Medicine and Biobehavioral Health, University of Minnesota Medical School, Duluth Campus, 1035 University Drive, Duluth, MN 55812 USA; 2 Department of Obstetrics, Gynecology and Women’s Health, University of Minnesota Medical School, Minneapolis, MN USA; 3 Department of Biomedical Sciences, University of Minnesota Medical School, Duluth Campus, Duluth, MN USA

**Keywords:** Abortion education, Medical education, Bioethics, Curriculum

## Abstract

**Purpose:**

Despite the frequency of abortions, one-third of medical schools in the US and Canada did not include coverage of that topic, according to a survey conducted in 2002–2005. The purpose of this project was to develop, implement, and evaluate a module for second year medical students related to the ethics of abortion.

**Methods:**

The module was designed as Independent Learning Time (ILT). The stated purpose was for students to consider some of the recent debate in the ethics literature related to conscientious objection and abortion and how personal views may influence future practice. The ILT included readings and Power Points to view. Students were asked to write a one-page reflection on one of three writing prompts.

**Results:**

The most commonly selected writing prompt in three classes was on personal values in relation to abortion (56.5%), followed by information about nearest provider of reproductive services to rural preceptor site (34.7%), followed by conscientious objection (23.3%). We received many positive comments about the ILT, including: “First, I would like to acknowledge my gratitude for this assignment and its subject. I believe it is very important that future physicians learn the entirety of women’s reproductive health care, including abortion and contraception, but unfortunately this is not always the case in medical training”.

**Conclusions:**

There has been an extremely positive response to the ILT. With the exception of the prompt specific to our regional campus mission that includes rural preceptorships during the preclinical years, this module could be implementable at other medical schools.

**Supplementary Information:**

The online version contains supplementary material available at 10.1186/s12978-023-01686-w.

## Background

In the US, abortion is common, with a rate of 11.2 per 1000 women aged 15–44 years in 2020 [1]. Despite the frequency of abortions, one-third of medical schools in the US and Canada did not include teaching about abortion, according to a survey conducted in 2002–2005 [2]. With the Dobbs Supreme Court decision in 2022, there has been renewed concern about abortion education in medical training [3] and a call for conscientious provision of services along with existing conscientious refusal [4].

The organization Medical Students for Choice (MSFC) “believes that abortion and family planning training should be a standard part of all medical school curricula” [5]. Specific to ethics of abortion and physician responsibility, MSFC includes the following learning objective: Understanding of the ethical and legal issues surrounding abortion and physician responsibility. In group interviews of MSFC members affiliated with 7 MSFC chapters in 4 Midwestern states, students related absence of abortion in the medical school curriculum and suggested that abortion education be provided in both the preclinical curriculum and in the obstetrics and gynecology (OB-GYN) clerkship [6]. A survey of OB-GYN clerkship directors published in 2005 found that 25% included no formal education about abortion and 23% reported that they did not know if formal education about abortion was included in the preclinical years [7]. There was no mention of informal education.

Best practices for teaching abortion content to medical students have not been developed and no curriculum has been published to date, but King and Penzias advocate for the following when teaching about abortion and other morally and spiritually charged topics: listen, commit to an open discussion and create a supportive environment [8]. Burns and Shaw noted that abortion education is lacking for medical students and proposed Observed Structured Clinical Examination (OSCE) to assess sensitive and nuance discussion with patients about pregnancy options [9].

The University of Minnesota Medical School, Duluth campus, was founded in 1972 with a mission to be a leader in educating physicians dedicated to family medicine, to serve the needs of rural Minnesota and Native American communities. Courses during the first two, pre-clinical years are systems-based and taught in blocks. Hormonal and Reproductive Medicine is a six-week course taught toward the end of the second year. Student evaluations at the end of the course in 2019 included a request that information related to ethics of abortions be added to the curriculum. An independent learning module on the ethics of abortion was developed and implemented the following year, and then revised based on student feedback for the next year. Student feedback and response to the ILT is included for the first three years of implementation. Separately in the curriculum, students are introduced to medication and surgical abortion care. This manuscript summarizes course development and implementation of this independent learning module in the first three years of content delivery. The project was reviewed by the University of Minnesota Institutional Review Board with a determination that it was not human subjects research.

## Methods

This is a descriptive, cross-sectional evaluation of medical school curriculum. The aim of the study was to develop, implement, and evaluate a new independent learning module on the ethics of abortion for second year medical students.

### Curriculum development and implementation

The Ethics of Abortion module was designed as an Independent Learning Time (ILT) assignment, with 110 min blocked in the schedule for completion, and a due date 3 ½ weeks after the assignment and materials were released in the student calendar. The curriculum design team included an OB-GYN clerkship director (SLH), the course director for Hormonal and Reproductive Medicine (AKS), and a certified healthcare ethicist (CAM). All team members were involved in all three years of development, implementation, and evaluation. The stated purpose of the assignment was: for students to consider some of the recent debate in the ethics literature related to conscientious objection and abortion and how personal views may influence future practice. Legal standing about abortion was not included, with students advised during a live session to stay tuned to Federal, state and county legislation regarding abortion. A course on law and medicine prior to the course on hormonal and reproductive medicine covered the details of abortion legislation in the US.

Learning objectives for the abortion ethics ILT included the following: (1) learners will compare stances from professional organizations related to abortion care, (2) learners will understand the arguments for and against conscientious objection for abortion care/referral, and (3) learners will explain how their personal views toward abortion will intersect with their future patient care as a physician.

Learning activities included: (1) reading a chapter in a book by a physician who had changed his stance on providing abortion care to his patients, (2) read four medical professional organization stances on abortion care, (3) view two Power Points on abortion, and (4) read two articles that provide a point/counter point to a duty to refer and conscientious objection.

Supplementary, non-required, resources were provided for additional reading. Full details about the module are available in the Appendix.

Assessment was done via reflection. Students were asked to choose one of three prompts and to write a one-page reflection. The three writing prompts were:

1. Where is the nearest provider of abortion services to your RMSP site? Does the local hospital at your RMSP site provide tubal ligations and vasectomies? How would this availability of reproductive services and your own personal beliefs influence your practice? (RMSP refers to Rural Medical Scholars Program, a required course for medical students on the Duluth campus in support of the rural mission. Students have 5-weeks of clinical placements at rural sites in Minnesota and western Wisconsin during their first two years of medical school).
2. Dr. Parker is a Christian and frames his argument to support reproductive choice as a moral one. Explore your own values in the context of your upbringing and how they influence your opinion of abortion services.
3. What is your opinion of conscientious objection and referral for abortion services?

The grading rubric for the reflection can be found in the appendix. This rubric is used for other reflection assignments in the pre-clinical curriculum. One person (CAM) graded all of the reflections. Grading for the Hormonal and Reproductive Medicine course is pass/fail where students must obtain 70% of total course points to pass the course. This assignment was worth 2.8% of the total grade.

Evaluation of the reflection component was undertaken by assessing the distribution of students choosing the three available writing prompts and the number of students asked to revise their reflection for further engagement if initial scores were less than 6. Comments received from students were grouped into positive comments and constructive feedback that could be considered for incorporation into the curriculum the following year.

## Results

The Ethics of Abortion ILT has been completed by three cohorts of second year medical students: 63 in the first year that it was implemented (2020), 66 in the second year (2021), and 64 in the third year (2022). Demographics of the cohorts include: 15 (7.7%) first generation students, 69 (35.4%) disadvantaged, 34 (17.4%) multicultural, and 114 (58.5%) who identified as women.

**Fig. 1:**
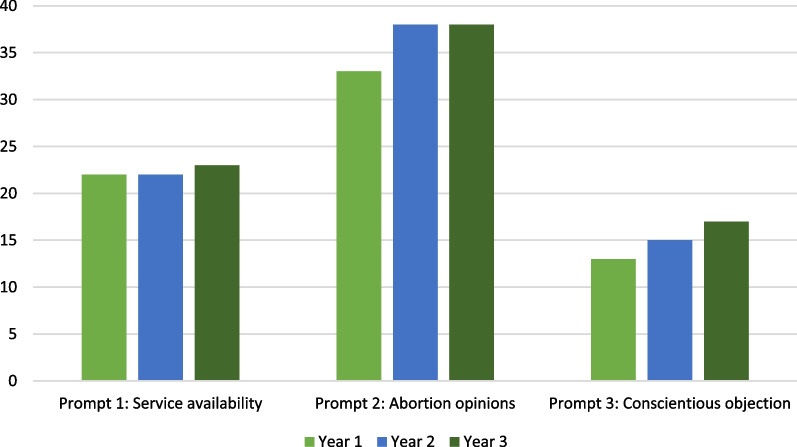
Distribution of selection of three reflection prompts

The distribution of students who responded to the three reflection prompts can be found in Fig. 1. Note that the numbers sum to greater than the total number of students because some students responded to more than one of the prompts. No students were invited to revise their reflections.

A number of students included unsolicited positive comments about the ILT along with their reflections (3 in 2020, 6 in 2021, and 3 in 2022). Some illustrative comments included the following:

 I really appreciate the time in our medical school curriculum that is dedicated to continued reflection on this topic, I think it is really important prior to going into clerkship years.

 I’m glad we have had the time to reflect on a hotly debated ethical issue within the bound of this class while also not directly meeting with our peers. I am not sure how this has been conducted in the past, but as this is an emotional ethical issue, I think it’s been good to have had the space to reflect while not openly confronting it without peers around us. I am sure this will be a challenging topic for all of us in many ways as we develop throughout our time as physicians, and even into our retirement. The fact that this is a difficult discussion demonstrates that it matters, and that it matters a lot.

 I found this assignment to be one of great insightfulness and reflection.

 I would like to start by mentioning that I’m grateful that the topics of reproductive health and reproductive justice have been incorporated into our medical school curriculum, and I appreciate the safe space that our school allows to discuss these emotionally and politically charged topics. These topics have historically been highly controversial–perhaps now, more than ever, with our current political climate–and they’re somewhat taboo to talk about. In order to achieve more favorable health outcomes for everyone involved, it’s important to foster open discussion about the topic and practice active listening when hearing from both sides of the debate.

 First, I would like to acknowledge my gratitude for this assignment and its subject. I believe it is very important that future physicians learn the entirety of women’s reproductive health care, including abortion and contraception, but unfortunately this is not always the case in medical training.

Critical feedback that was received came from three students in student evaluations about the module from the anonymous final course evaluation. These are the critical comments that were received in the first 3 years of module implementation:

- Although I am sure this was not the intention, in my perspective, it felt that assigning this article to read was using Dr. Parker’s “Christian background” as leverage to specifically target students who identify as Christian, singling them out, and using this chapter as an attempt to undermine the very basic teachings of the Christian faith as a way to proclaim that abortion can be justified and is in fact a “Christian act.” Additionally there was zero content to ask students to ethically consider the negative impacts of abortion in our culture.
- I am SO glad this was included in the curriculum, but I think it would be really beneficial to have more of a required small group discussion about abortion in addition to the reflection- much like we did for physician aid in dying in the End of Life course. I appreciate the reflection, but I think it would be really helpful for the class to hear other perspectives in real time instead of reading a paper and reflecting… All in all, I’m glad abortion is at least somewhere in the curriculum, a topic that I recognize is controversial and not included in all medical school curricula, but I would have loved to see it less as a self-directed learning opportunity.
- If you’re going to do this assignment please put it out in a way that actually represents both sides equally and fairly. The way most of these articles read they seemed to be presenting one side as clearly immoral and wrong and not representing what they actually believe.

## Discussion

This Ethics of Abortion ILT was developed in response to medical student request for abortion education in the Hormonal and Reproductive medicine block in year 2. There has been an extremely positive response to the ILT, with several students indicating that, not only were they grateful to have the opportunity to reflect on the topic, but also that they were able to do that in private due to the controversial nature of the topic. The fact that the majority of students chose the reflection prompt related to personal values associated with abortion further supports this positive student response to the module and interest in considering personal views about abortion.

Having this curriculum in the preclinical years helps provide a space for students to consider this topic prior to starting on clinical rotations like OBGYN and family medicine where they will treat patients considering abortion and to have already had exposure to this topic in the preclinical years, and to consider this very polarizing medical service and possibly increase mindfulness in the care of pregnant patients. In Minnesota, it helps to prepare students for the ethics discussion that occurs in the OBGYN clerkship that encompasses a case on abortion and family medicine and OBGYN rotations where discussion of abortions may occur and consideration of the ethical principles in relation to abortion from the physician and patient standpoint.

We are not aware of any other published curriculum on this topic for preclinical medical students. With the use of interviews with fourth year medical students who had applied to OB-GYN residency programs, researchers discovered that the term “elective” in relation to abortion education reflects personal, judgmental bias [10], and that many students were committed to providing or referring for abortion because they felt that it was the ethically correct thing to do [11].

For faculty wanting to incorporate this module into their curriculum, a possible change would be to delete the first prompt about availability of reproductive services at their local rural preceptor site if that is not relevant. Given the smaller percentage of students choosing the prompt about conscientious objection, that prompt could also potentially be deleted, with just the single prompt about attitudes toward abortion offered as a reflection. Small group discussion or case simulation could also be considered, with assurance of psychological safety and experienced small group facilitators, and balance with expressed desire by some students to initially process this material in private.

## Conclusions

In conclusion, we have demonstrated a highly successful ethics of abortion independent learning module that was developed and revised in response to medical student request. With the exception of the prompt specific to our regional campus mission of rural health that includes rural preceptorships during the preclinical years, this module should be immediately implementable at other medical/health professional schools. This module may also be relevant to residents in OB/GYN, family medicine, internal medicine, and emergency medicine where care for patients deciding what to do about pregnancy will be encountered.

### Supplementary Information

**Additional file 1.** Ethics of abortion.

## Data Availability

Not applicable.
