# Supplementary material for: “It’s important to foster open discussion about the topic”: development, implementation, and evaluation of an ethics of abortion independent learning module for second year medical students
Source: Reprod Health. 2023 Sep 25;20:145. doi: 10.1186/s12978-023-01686-w (PMC10521547; doi:10.1186/s12978-023-01686-w)
Supplement: Supplementary file 1 — Additional file 1. Ethics of abortion. [file 12978_2023_1686_MOESM1_ESM.docx]

**Ethics of Abortion – Reflection Assignment**

The purpose of this assignment is for students to consider some of the recent debate in the ethics literature related to conscientious objection and abortion and how personal views may influence future practice.

**Learning Activity and Grading**

- Specific learning objectives include the following:
  - Learners will compare stances from professional organizations related to abortion care.
  - Learners will understand the arguments for and against conscientious objection for abortion care/referral
  - Learners will explain how their personal views toward abortion will intersect with their future patient care as a physician
- Read Chapter 1 in: Life’s Work. A Moral Argument for Choice by Dr. Willie Parker. Published by Simon and Schuster, 2017.
- Read professional organization stances on abortion care:
  - AMA stance: “A physician shall, in the provision of appropriate patient care, except in emergencies, be free to choose whom to serve, with whom to associate, and the environment in which to provide medical care” (AMA Code of Medical Ethics, 2001). “AMA Code of Medical Ethics Available Online.” AMA Journal of Ethics, vol. 3, no. 4, 2001, doi:10.1001/virtualmentor.2001.3.4.code1-0104.
  - AMA announces new adopted policies related to reproductive health care, Nov 2022: <https://www.ama-assn.org/press-center/press-releases/ama-announces-new-adopted-policies-related-reproductive-health-care>
  - Code of Medical Ethics Opinion 4.2.7: The Principles of Medical Ethics of the AMA do not prohibit a physician from performing an abortion in accordance with good medical practice and under circumstances that do not violate the law.
  - ACOG Abortion Policy: “While ACOG recognizes and respects that individuals may be personally opposed to abortion, health care providers should not seek to impose their personal beliefs upon their patients nor allow personal beliefs to compromise patient health, access to care, or informed consent.”
  - The Catholic Ethical and Religious Directives for Healthcare Services, Sixth Edition. Directive 45: Abortion (that is, the directly intended termination of pregnancy before viability or the directly intended destruction of a viable fetus) is never permitted. (<https://www.usccb.org/resources/ethical-religious-directives-catholic-health-service-sixth-edition-2016-06_0.pdf>)
  - World Medical Association (the American Medical Association is a member) opinion on mandatory referral: *During the debates, the most contentious proposal concerned mandatory referral, obliging doctors who object to controversial procedures such as physician assisted suicide or abortion to refer to a willing doctor. After considerable debate, a compromise agreement was reached that does not require doctors to refer in case of a conscientious objection. Rather, the compromise says ‘Physician conscientious objection to provision of any lawful medical interventions may only be exercised if the individual patient is not harmed or discriminated against and if the patient’s health is not endangered.* (<https://www.wma.net/news-post/physicians-code-of-medical-ethics-updated/>)
- View the following Power Points:
  - Abortion in the US after legalization by Carolyn Joffe, PhD, UCSF, 27 minutes
    - <https://www.innovating-education.org/2016/02/abortion-in-the-us-after-legalization/>
  - Abortion and Its Multiple Contexts by Carolyn Sufrin, MD, PhD, UCSF, 24 minutes
    - <https://www.innovating-education.org/2016/02/abortion-in-multiple-contexts/>
- Read the following two articles that provide a point/counter point to a duty to refer and conscientious objection:
  - Cowley C. Conscientious objection in healthcare and the duty to refer. J Med Ethics 2017;43:207-212. PMID: 28255026. https://jme-bmj-com.ezp1.lib.umn.edu/content/43/4/207
  - Finegan T. Conscientious objection to referrals. PMID: 30242077. J Med Ethics 2019;45:277-279. https://jme-bmj-com.ezp1.lib.umn.edu/content/45/4/277
- Supplementary readings are listed in additional resources, but are not required.
- Select one of three questions for reflection and respond in a maximum of one page.
- Assignments will be graded according to the rubric, with score contributing to overall course grade as per syllabus.

**Assignment due:** Midnight, 3 ½ weeks after assignment and materials released to students

**Additional Resources:**

Blackshaw BP, Rodger D. Questionable benefits and unavoidable personal beliefs: defending conscientious objection for abortion. J Med Ethics 2019;0:1-5. PMID: 31473656

Bonow A, Nokes E, eds. Shout Your Abortion. PM Press, 2018. Interview with four providers start on page 173.

Fiala C, Arthur JH. There is no defense for “conscientious objection” in reproductive health care. Eur J Obstet Gynecol Reprod Biol 2017;216:254-258. PMID: 28757115

Gerrard JW. Is it ethical for a general practitioner to claim conscientious objection when asked to refer for abortion? J Med Ethics 2009;35:599-602. PMID: 19793938

McLeod C. Conscience in Reproductive Health Care. Prioritizing Patient Interests. Oxford University Press, 2020.

Morrell KM, Chavkin W. Conscientious objection to abortion and reproductive healthcare: a review of recent literature and implications for adolescents. Curr Opin Obstet Gynecol 2015;27:333-338. PMID: 26241174

Safe and Legal Abortion Options in Minnesota. Minnesota Monthly. June 28, 2022. <https://www.minnesotamonthly.com/lifestyle/safe-and-legal-abortion-options-in-minnesota/>

Weiner R. Conscientious objection: a Talmudic paradigm shift. J Relig Health 2020; 10 Jan. PMID: 31925633.

**Choose from one of the following three questions for reflection (one page maximum).**

1. Where is the nearest provider of abortion services to your RMSP site? Does the local hospital at your RMSP site provide tubal ligations and vasectomies? How would this availability of reproductive services and your own personal beliefs influence your practice?
2. Dr. Parker is a Christian and frames his argument to support reproductive choice as a moral one. Explore your own values in the context of your upbringing and how they influence your opinion of abortion services.
3. What is your opinion of conscientious objection and referral for abortion services?

**Grading Rubric**

| 0 points | 2 points | 4 points | 6 points | 8 points |
| --- | --- | --- | --- | --- |
| Assignment not turned in (or turned in late) | Shows minimal engagement  with the topic, failing to  recognize multiple  dimensions/ perspectives;  lacking even basic  observations | Shows some engagement with  the topic without elaboration;  offers basic observations but  rarely original insight | Demonstrates engagement  with the topic, recognizing  multiple dimensions and/or  perspectives; offers some  insight | Demonstrates engagement  with the topic, recognizing  multiple dimensions and/or  perspectives with elaboration  and depth; offers considerable  insight |
